# ‘Pneumonia has gone’: exploring perceptions of health in a cookstove intervention trial in rural Malawi

**DOI:** 10.1136/bmjgh-2020-004596

**Published:** 2021-10-11

**Authors:** Jane Ardrey, Kate Jehan, Caroline Kumbuyo, Chifundo Ndamala, Kevin Mortimer, Rachel Tolhurst

**Affiliations:** 1 Department of International Public Health, Liverpool School of Tropical Medicine, Liverpool, UK; 2 Department of Public Health, Policy and Systems, Institute of Population of Health Sciences, University of Liverpool, Liverpool, Merseyside, UK; 3 Malawi-Liverpool-Wellcome Trust Clinical Research Programme, Blantyre, Malawi; 4 Department of Clinical Sciences, Liverpool School of Tropical Medicine, Liverpool, UK

**Keywords:** environmental health, pneumonia, respiratory infections, qualitative study

## Abstract

**Introduction:**

Air pollution through cooking on open fires or inefficient cookstoves using biomass fuels has been linked with impaired lung health and with over 4 million premature deaths per annum. However, use of cleaner cookstoves is often sporadic and there are indications that longer-term health benefits are not prioritised by users. There is also limited information about how recipients of cookstoves perceive the health benefits of clean cooking interventions. We therefore conducted a qualitative study alongside the Cooking and Pneumonia Study (CAPS).

**Methods:**

Qualitative methods and the participatory methodology Photovoice were used in an in-depth examination of health perceptions and understandings of CAPS trial participants. Fifty participants in five CAPS intervention villages collected images about cooking. These were discussed in village-level focus groups and in interviews with 12 representative participants. Village community representatives were also interviewed. Four female and eight male CAPS fieldworkers took part in gender-specific focus groups and two female and two male fieldworkers were interviewed. A thematic content approach was used for data analysis.

**Results:**

We found a disconnect between locally situated perceptions of health and the biomedically focused trial model. This included the development of potentially harmful understandings such as that pneumonia was no longer a threat and potential confusion between the symptoms of pneumonia and malaria. Study participants perceived health and well-being benefits including: cookstoves saved bodily energy; quick cooking helped maintain family harmony.

**Conclusion:**

A deeper understanding of narratives of health within CAPS showed how context-specific perceptions of the health benefits of cookstoves were developed. This highlighted the conflicting priorities of cookstove intervention researchers and participants, and unintended and potentially harmful health understandings. The study also emphasises the importance of including qualitative explorations in similar complex interventions where potential pathways to beneficial (and harmful) effects, cannot be completely explicated through biomedical models alone.

**Key questions**

**What is already known?**

- There is evidence that household air pollution from the burning of solid fuels in open fires and inefficient cookstoves is detrimental to health.
- Cleaner burning cookstoves are available but are often used intermittently or in combination with less clean alternatives.
- The drivers of adoption of cleaner cookstoves are multifactorial and embedded in gendered household and community dynamics, with longer-term improved health seldom prioritised.

**What are the new findings?**

- A qualitative and participatory approach led to a rich understanding of locally situated health-based priorities, which differed from the biomedical health messages of the trial.
- The development of ‘syncretic’ understandings of the link between pneumonia and cookstoves, possibly inadvertently communicated through trial messaging, may have potentially harmful unintended consequences.
- Users perceived the cookstoves as ‘healthier’ because they saved strength when lighting and tending, and because quick cooking facilitated family harmony.

**What do the new findings imply?**

- An important implication of these findings is that health interventions and trials may result in unexpected and potentially harmful health understandings.
- The health priorities of the implementors of technological interventions and those of users, may differ considerably.
- Gaining a deeper understanding of biosocial perceptions of health and well-being within interventions using qualitative and participatory methodologies, can promote the health priorities of research participants and militate against negative health impacts.

## Introduction

Household air pollution is a multisectoral issue linked with several Sustainable Development Goals (SDGs) with specific targets relating to clean energy in homes, improving air quality and a reduction in air pollution-related deaths and illness.^1^ It is estimated that in 2019 household air pollution (HAP) from burning biomass fuels primarily for cooking, contributed to 2.31 million deaths.^2^ The use of open fires or inefficient cookstoves for cooking is widespread in many low-income and middle-income countries (LMICs)^3^ including in Malawi where 98% of the population cook using biomass fuels.^4^

HAP can lead to harmful and irreversible effects on the lung and other health systems.^5^ As the development of lung and immune systems occurs both prenatally and throughout early life, exposure to air pollution at these times can be particularly detrimental.^5^ HAP has also been linked with respiratory infections in older children and adults which are leading causes of morbidity and mortality especially in LMICs.^3^ Pneumonia in under-5s accounts for 15% of all deaths in this age group.^6^ Children and their mothers can be greatly exposed to HAP where cooking using biomass fuels takes place.^7^

The use of cleaner burning cookstoves has been proposed as a solution to HAP by advocacy organisations such as the Clean Cooking Alliance with suggested benefits including environmental remediation and improved health.^8^ The Cooking and Pneumonia Study (CAPS) was carried out in rural Malawi from 2013 to 2016 with the aim of testing the hypothesis that the considerable burden of pneumonia in under-5s in this setting, could be attributable to HAP from open fire cooking. Two cleaner burning biomass cookstoves were given to households in intervention villages and control village residents continued to cook on open fires.^9^ The trial found that there was no evidence of a reduction of pneumonia in the intervention group. Among the multifactorial reasons for this result, it was suggested that sustained use of any cleaner cooking interventions, whether cookstoves or fuels would need to ‘achieve a high level of acceptance’ in order for health benefits to be realised.^9^ As Gordon *et al* describe, the use of old and new technology alongside one another (so called ‘stacking’), and a reduction or stopping of clean cookstove use over time presents significant challenges to clean cooking interventions generally.^3^

Within clinical trials and most health-based research, a biomedical model of health predominates. However, this biomedical framing is not the sole or even the dominant framing of health among lay populations. For example, in Greco *et al*’s exploration of well-being among rural Malawian women, the authors found that locally defined quality of life measures extended beyond the material and were ‘highly dependent on complex feelings, relations and social norms’ that were vividly expressed by study participants.^10^ In addition, there can be important differences between the health perceptions and understandings of health research participants, and the researchers who instigate and conduct trials.^11 12^

Social relationships within trials are likely to be influential but generally under-researched or under-recognised in health research and policymaking.^13 14^ One way to explore these differences is through the lens of ‘medical syncretism’ as described by Muela *et al*,^15^ which suggests that biomedical and local concepts of health might be blended and merged to create understandings that may differ from those intended.

There also appears to be a mismatch between the emphasis on the health benefits of cookstoves as promoted by researchers and implementors and the priorities of cookstove users.^16 17^ As described above, there are indications that cookstove users living in difficult circumstances may prioritise other useful factors beyond longer-term ‘invisible’ health impacts. However, little is known about how cookstove users perceive the impact of cookstoves on their health. In this study, qualitative methods and the participatory methodology Photovoice were used to explore understandings of health within a large-scale cookstove intervention, with the aim of encouraging a greater emphasis on the health and well-being of cookstove intervention recipients in low-income settings.

Qualitative methods are often used as part of large-scale clinical trials, to explore in more detail the perceptions of participants and in recognition of the complexity of the social contexts in which the research takes place.^18^ In a mixed-method systematic review of barriers and enablers to uptake of improved cookstoves, Rehfuess *et al* found 31 factors that influence uptake but concluded that while some are more important than others, none can ensure sustained adoption.^19^ Stanistreet *et al* carried out an in-depth analysis of the 14 qualitative studies identified, with the aim of exploring views of cookstove users and stakeholders on these factors.^20^ They concluded that qualitative research ‘can provide a voice for the stove user’ especially regarding contextual decision-making about cookstove use.^20^

While recognising the value of qualitative research in exploring perceptions of health in a cookstove intervention, the aim of this study was to move away from the dichotomy of barriers and enablers, in order to challenge existing ‘hierarchies of knowledge’^21^ in global health, including biomedical health narratives. In addition, the use of qualitative and participatory methodologies acknowledges that randomised controlled trials such as CAPS have ‘deep social implications’^14^ including the development and shifting of understandings of health. Photovoice is rooted in the emancipatory thinking of Paulo Freire, feminist research methodology and inclusive documentary photography, and like other participatory methodologies, it recognises that all people are sources of expertise.^22^ The methodology was initially designed to explore perceptions of health^23–25^ and has been used in many contexts; examples in Malawi include a study of well-being of palliative care patients^26^ and adolescent well-being.^27^ There has, however, been limited use of the methodology in relation to cookstove interventions, exceptions being the pilot work for this study^28^ and a study with potential users of portable gas cookstoves in Cameroon.^29^

Qualitative methods and the participatory methodology Photovoice were therefore combined in this study with the overall aim of gaining a deeper understanding of the sociocultural context of cookstove use within CAPS, and the implications of this for future implementation of clean cooking initiatives. One of the original research objectives was to explore the perceived and experienced benefits of using improved cookstoves from the perspectives of trial participants and fieldworkers. This and the other objectives were changed in response to inductively derived findings to develop three research questions. In this manuscript we report on health-related findings that respond to the following question: ‘How do CAPS participants experience the trial and how is this linked with understandings of health, technology, and the research process?’. Other findings will be reported elsewhere.

## Methods

### Study setting and design

This study was carried out in Chikwawa in rural Malawi. In 2018, the World Bank estimated that over 50% of the Malawian population were living in poverty due to limited economic opportunities and reliance on low productive agriculture.^30^ The country has a high disease burden and leading causes of mortality include childhood pneumonia^31^ and non-communicable diseases.^32^ Almost all of the population, 98% in 2015,^4^ cook using solid fuels and HAP exposure is widespread. The Malawi-Liverpool-Wellcome Trust Clinical Research Centre (MLW) has carried out research in Chikwawa since 2002.^33^ Their Chikwawa site office was one of two bases for the CAPS conducted between 2013 and 2016^9^ and for this nested qualitative study. A phased qualitative research study was carried out between April and November 2016 using observation, focus groups, interviews and the participatory methodology Photovoice.

Photovoice was used in this study both in acknowledgement of the effectiveness of the methodology in elucidating the hidden in the everyday practice of cooking,^34^ but also in recognition of the expertise of cooks in Chikwawa and their valuable experiential knowledge.^35^ The experience of the first author when piloting the methodology within CAPS, indicated that Photovoice had the potential to facilitate a deep exploration of the everyday realities of participant’s lives and to militate against the social desirability bias that might be anticipated in this type of ‘nested’ study.^34^ Social desirability bias is the tendency of research participants to present themselves favourably^36^ and within CAPS could potentially have led to overly positive reporting of cookstove benefits. Visual methodologies such as Photovoice have been shown to shift the focus of the research encounter towards participants who collect and discuss images that reflect their own concerns.^24 37 38^

As delineated by the originators of Photovoice methodology, the focus was on the definition and interpretation of images by participants and not the images themselves.^24^ Photovoice activities therefore included focus group discussions (FGDs) and interviews. In the Photovoice FGDs the SHOWeD acronym employed by Wang and Burris, that is: what do you See here?; what’s really Happening here?; how does this relate to Our lives?; Why does this problem or this strength exist?; what can we Do about this?^39^ was incorporated into the study design to encourage wide ranging, open discussion.

**Figure 1:**
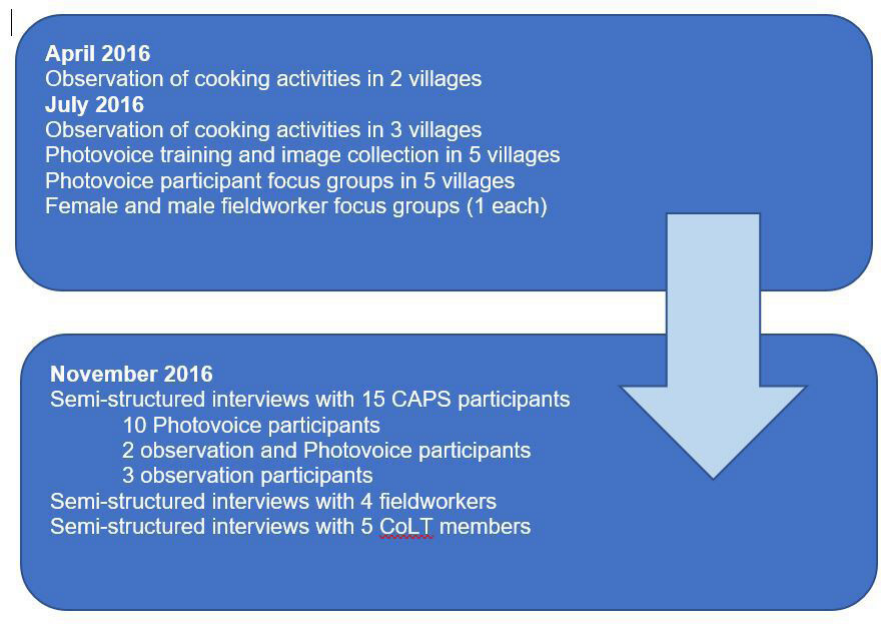
Overview of timeline, methods, methodology and participants. CAPS, Cooking and Pneumonia Study; COLT, Community Liaison Team.

In summary, this was a qualitative study in which methods of observation, FGDs and interviews were blended with the participatory methodology Photovoice in a flexible and iterative study design developed over time, with three phases in April, July and November 2016 as shown in figure 1.

### Study participants

The study sample included three groups: CAPS trial participants, fieldworkers and Community Liaison Team members (CoLTs). The role of the CoLT within MLW is similar to that of other Community Advisory Groups in comparable settings. CoLTs are residents of places where MLW carry out research and an important part of their role is to identify and feedback the concerns of research participants to MLW.^40^ CAPS trial participants were from five intervention villages. The study villages were purposively selected as representative of the wider CAPS cohort using factors such as size, availability of facilities such as school and clinics and access to trading centres.

The main selection criterion for Photovoice participants was gender; eight women and two men from each village were recruited in recognition of the primary role of women as family cooks and of the unequal intrahousehold power of men, in this context.^41^ This sample was also purposively selected to include a range of ages, female heads of households and non-users of cookstoves. Each village had an assigned CoLT member who was recruited for this study. They were all in their 40s, married with children and had been CoLT members for some years; two were women and three men. CAPS fieldworker participants were symbolically representative of the wider fieldworker group. That is, although they participated voluntarily and were not purposively selected, the group represented a range of ages, experience, seniority and gender. There were 12 fieldworkers recruited, eight were men and four women which reflected the existing unequal gender mix. From these 12, four fieldworkers were interviewed, two women and two men; they were all in their 30s and had been CAPS fieldworkers from the outset of the study.

In each of the five selected villages, the following activities were carried out: observation of a cooking session; Photovoice training, image collection and focus group; semi-structured interviews with three Photovoice and one observation participant. The latter designation refers to the women that prepared a meal while being observed by the CAPS Qualitative Research Assistant (CK) and the first author (JA). In two of the villages the observation participant was also a Photovoice participant. The CoLT members for each village were interviewed and asked: to share their views on how CAPS participants viewed the trial and whether it had beneficial or negative impacts; about family structures in Chikwawa and how family members spent their time; what is good health for someone living in Chikwawa. Eight male fieldworkers and four female fieldworkers participated in gender-specific FGDs in which they discussed any differences between CAPS and other research, whether CAPS was beneficial for participants and barriers and motivators of adoption of the cookstoves. From those groups, two male and two female fieldworkers were interviewed, to explore more deeply the potential benefits of cookstoves, how this may be linked with gendered household roles, illiteracy and religious beliefs and what fieldworkers thought participants understood by the term pneumonia.

The Photovoice element of this study was informed by pilot work described elsewhere.^34^ In each selected village, there were three stages in the Photovoice process. First, Photovoice participants were trained in the use of a digital camera by a local photographer and advised about potential problems that may be encountered, with an emphasis on the avoidance of risk. CK then explained the aim of the Photovoice process and facilitated a discussion of the ethical collection of images. Each participant was asked to collect 50 images over 5 days showing: what they ate; how this food was cooked; who cooked the food; who they cooked and ate with. Participants were informed that they could take 20 images for themselves to keep.

**Figure 2:**
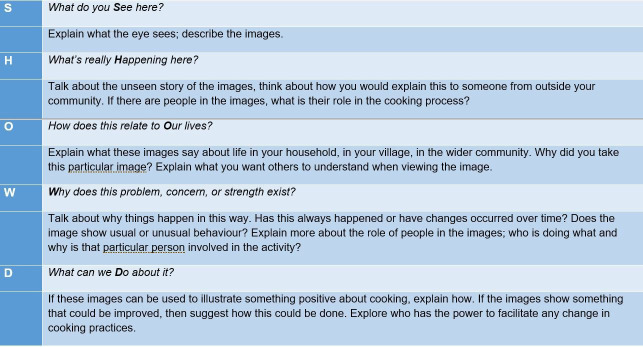
Photovoice focus group discussion SHOWeD questions.

**Figure 3:**
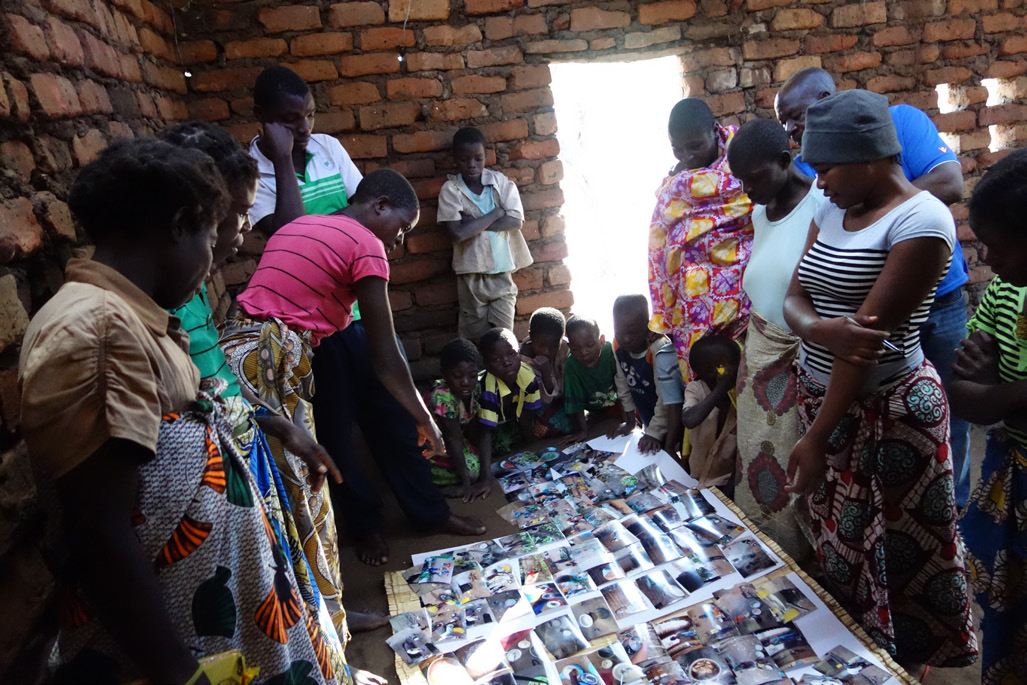
Photovoice focus group discussion in progress in Village 2.

After 5 days, the cameras were collected and taken to the nearest town to be processed. The first 75 images on each camera were printed off and distributed to the photographers at village level focus groups. FGDs took place in local village facilities such as schools or communal buildings. The images selected for discussion by the participants were spread out on large sheets of paper on the floor and grouped into discussion themes, in line with the process of selecting and contextualising of images recommended by Wang and Burris.^22^ Practically, participants selected the images they wanted to discuss and began the contextualisation by grouping together images related to a specific topic. Example discussion themes included types of cooking apparatus such as open fires and types of cookstoves but also related to the growing, preparation and sharing of food. An amended version of the SHOWeD acronym^39^ was used as shown in figure 2 to encourage deeper discussion of the story behind the images. Figure 3 shows a Photovoice FGD in progress in Village 2 with participants gathered around grouped images so that all had the opportunity to view and contribute to the discussion

Finally, in November 2016, semi-structured interviews were carried out with selected Photovoice participants and the observation participants, informed by interim analysis. Interviewees were asked: about food they had cooked the previous day including where it was obtained, how it was cooked and who shared it; to reflect on any health impacts of the intervention cookstoves; and how any saved time from faster cooking was used.

Demographic details of Photovoice participants are in table 1.

**Table 1:** Photovoice participant demographic details

Village	Gender	Age range	List of participant occupations
1	8 female 2 male	24–32 28–50	Bicycle hire; casual labourer; subsistence farmer (7 as primary occupation and 3 as secondary).
2	8 female 2 male	20–65 23–32	Bicycle hire; casual labourer; subsistence farmer (7 as primary occupation and 3 as secondary).
3	8 female 2 male	23–38 46–51	Sells doughnuts; sells maize; builder; guard; subsistence farmer (6 as primary occupation and 2 as secondary); pastor.
4	8 female 2 male	18–35 30–36	All—primary occupation of subsistence farmer; shop owner; charcoal burner.
5	8 female 2 male	20–49 23–53	Sells doughnuts; shop owner; pump attendant; sells phones; commercial farmer; subsistence farmer (6 primary occupation and 3 as secondary).

An overview of the timeline, methods/methodology used and participants, is shown in figure 1. The first author (JA) was present for and co-ordinated all activities with the exception of two of the fieldworker interviews. CK led on the selection of participants, conducted the remaining two fieldworker interviews and provided translation throughout.

### Data analysis

All focus groups and interviews were recorded using digital recorders and translation and transcription was carried out by CK and the MLW translation and transcription team. Thematic data analysis was completed by JA using the framework approach, a visual method using matrices to facilitate comparison of data.^42^ Data was coded inductively, and the same codes were applied across the data set. The following steps were undertaken: familiarisation with transcript content; coding of each transcript to develop a set of codes and subcodes; creation of a framework of all codes; summarising of data within framework; development of categories through collated codes; final mapping and interpretation. Inductive interim analysis was carried out between the July and November data collection periods to guide the development of interview topic guides. The framework analysis process facilitated the development of three research questions congruent with the overall study aim.

### Patient and public involvement

Photovoice participants were free to collect and discuss images that were important to them and therefore contributed to data collection and analysis. Each Photovoice village group also selected 10 images for exhibition and appointed two representatives who presented and discussed these at Photovoice exhibitions in the local community.

## Results

Analysis of data from this study suggests that there was a divide between CAPS participants’ largely local and social understanding of health, which emphasises well-being, and the biomedical messages that underpinned the CAPS trial and are inherent to much of MLW’s research. These local understandings of health can be described under three thematic areas. Theme 1, *definitions of good health in Chikwawa* provides the context for themes 2 and 3: *transformation of understandings of pneumonia* and *unanticipated health benefits of cookstoves*.

### Theme 1: definitions of good health in Chikwawa

Although CoLT members had a key role in the implementation of CAPS, including facilitating community meetings and fieldwork activities, when asked about good health in Chikwawa, they did not mention pneumonia or the potential health benefits of cookstoves. Instead they linked health with various factors specific to the local environment and particularly the prevalence of hunger linked with precarious livelihoods largely reliant on agriculture.

CoLT members described a healthy person as having a secure livelihood and access to food. That is, someone who:

is working, maybe on the farm, who has surplus food at his house. (CoLT member Village 1)

uses it [money] properly for their health…when buying your food, you should consider all six groups of food. (CoLT member Village 4)

On the other hand, an unhealthy person is described as follows:

[M]aybe we can say that the woman, children or the man, they may not be healthy as it is hard to obtain food in our area. (CoLT member Village 2)

Study participants described how people needed to be physically able to carry out work for them to obtain enough food for a healthy life. Owning land and livestock was also seen as important for promoting good health, as this provided the means to grow crops or rear animals that could be consumed or sold for profit. However, agriculture was also seen as potentially unreliable and multiple livelihood sources were recommended.

CoLT members also suggested that those who are not able to provide for their families are lacking in education; this is seen to lead to ‘ignorance’ and a healthy life is dependent on making informed decisions. Conversely, those who attend school are better able to plan their families and provide for their education and nutrition. Interviewees connected ill-health with being financially insecure and struggling to provide for families, as described below:

So, maybe we should say that the healthy life is that one which does not have another thought [that is, no worries] and also maybe your children are few, you have given birth to few children, then it means the food is enough at the home so then they say this lady is healthy. (female CoLT member Village 2)

Worry and ‘thinking too much’ were sometimes seen as linked with marital infidelity and leading to loss of appetite and hopelessness.

First, worries cause a person to be in bad health because you think too much…you cannot eat because you are thinking too much. For those in marriages it can be quarrels, maybe the husband or wife is having extra marital affairs so once they start thinking about that they think too much and even about hanging themselves. (male CoLT member Village 4)

In this way, CoLT members clearly described the pervasive impact of family discord as detrimental to health and well-being, linking this with the insecure food and livelihood context of Chikwawa, which provides daily challenges.

### Theme 2: transformation of understandings of pneumonia

As detailed in the introduction, CAPS did not result in a reduction in childhood pneumonia in study intervention villages. Despite this, when discussing the benefits of the trial, CAPS participants suggested that there had been a significant decrease in incidence of the disease. A female Photovoice participant said that:

With the coming of this research we saw that pneumonia has decreased…Yes, we can say that all of us present here can give testimonies…Yes, when all the women received these stoves, we have not heard of any children ill with pneumonia. (female CAPS trial participant Village 3 FGD)

CAPS fieldworkers provided some indication of why CAPS trial participants reported that pneumonia had gone. They played a crucial role in all trial processes in villages including communicating extensively with the participants about the possibility of a link between pneumonia and cookstoves. Although trial participants knew about childhood pneumonia and many would have had personal experience of its devastating impact, the potential link with cookstoves was a new factor.

Male fieldworkers who participated in the FGDs agreed that CAPS seemed ‘so scientific’ to the trial participants during the initial sensitisation stage and during field visits, suggesting that this information was not in their frame of reference and needed to be interpreted. They reported that CAPS participants rarely mentioned pneumonia, but one FGD member described how those trial participants ‘with a little bit of knowledge’ described how pneumonia was prevented because the cookstoves created a gas that then circulated through the house (male CAPS fieldworker). Others agreed that many CAPS participants formulate their own understandings and rationalisations of the link between cookstoves and pneumonia.

This issue was probed more deeply in the semi-structured interviews with CAPS trial participants. By this stage (November 2016), many of the cookstoves had stopped working due to problems with the battery or other maintenance issues. This resulted in a shift in the narrative with interviewees suggesting that pneumonia had come back after the cookstoves became defunct. They commonly referred to pneumonia ‘going’ and ‘coming back’, although it was not clear whether these descriptions were metaphorical (referring to a decline in pneumonia) or indicating a literal ‘distancing’ of pneumonia (through some mechanism of the cookstoves). When asked why pneumonia may have ‘gone’, interviewees reported their own experience of this phenomenon as in the example below:

[Now that use of cookstoves has been] abandoned, when going to the hospital…you will hear that children are being diagnosed with pneumonia. (female CAPS trial participant Village 5 interviewee)

The cookstoves were also linked with a reduction of other diseases including malaria and some participants suggested that receipt of the cookstoves alone (and not their use) led to these beneficial effects, as in this statement:

Ever since [we received the cookstoves] the child has never been diagnosed at the hospital with either pneumonia or malaria or any other disease. (female CAPS trial participant Village 4 interviewee)

In the CAPS fieldworker interviews, these ideas were explored further, and the response of interviewees indicates that existing understandings of pneumonia were changed through participation in CAPS. This included positive developments such as greater awareness of childhood pneumonia. However, there were also indications that existing ideas of pneumonia were merged with new understandings resulting in potentially negative consequences such as confusion between symptoms of pneumonia and other diseases, and delay in treatment seeking for pneumonia.

For example, a female fieldworker explained that through CAPS science communication activities, participants had more understanding of the extent of childhood pneumonia and that it can be life-threatening. A male CAPS fieldworker suggested that CAPS had resulted in earlier identification of pneumonia instead of at a later, more severe stage, where the child is ‘breathing fast and whistling’ (male CAPS fieldworker).

However, both male and female CAPS fieldworker interviewees also described how CAPS had resulted in cookstoves being associated with pneumonia. As described by a male fieldworker:

…the use of the word pneumonia in minds of the participants, I think the word was mostly used when they are talking to us …when we are [introducing the cookstoves] we are bringing the word pneumonia, I think there was little [knowledge about pneumonia before the trial] in the minds of participants.

A female fieldworker suggests that participants not only began to associate cookstoves and pneumonia, but also to link the intervention with prevention of disease generally, as below:

Because we said this is a study for cooking and pneumonia … they say this is the end of the pneumonia diseases once they receive the cook stove… [whereas some] take every disease as pneumonia, not the real pneumonia [that is, they confuse pneumonia with other diseases and associate the cookstoves with the decline of diseases in general].

In summary, CAPS resulted in the development of new understandings of pneumonia among trial participants. There were indications of potentially positive impacts through increased knowledge about symptoms. However, participants also reported that pneumonia had ‘gone’ because they had received cookstoves and appeared to link the receipt of cookstoves with improved disease management in general. Unanticipated and novel health benefits of cookstoves to CAPS participants were also identified and will be discussed in the next section.

### Theme 3: unanticipated perceived health and well-being benefits of cookstoves

CAPS trial participants, CoLT members and fieldworkers agreed that cookstoves were seen as healthier because it was not necessary to blow on them during lighting and throughout cooking. This view is clearly expressed in a CAPS participant Photovoice FGD as follows:

Those ones [participant points to a photo of an open fire] need us to blow air into, while the other ones blow themselves… we will just be taking our firewood and see that the fire is going down, then add in our firewood while that one [the cookstove], even when the firewood is ending, when we want to add the firewood, it will still blow it. (female CAPS trial participant Village 2 FGD)

In some cases, descriptions of this benefit include references to smoke inhalation and suggestions that ‘not blowing’ and reduced smoke were linked with the perceived reduction in pneumonia. For example, when asked why he thought pneumonia had declined, a CoLT member said:

I believe because the parents were now using the cookstoves, because they no longer blew the air. They no longer had difficulties to blow fire. There was no smoke. (male CoLT member Village 4)

**Figure 4:**
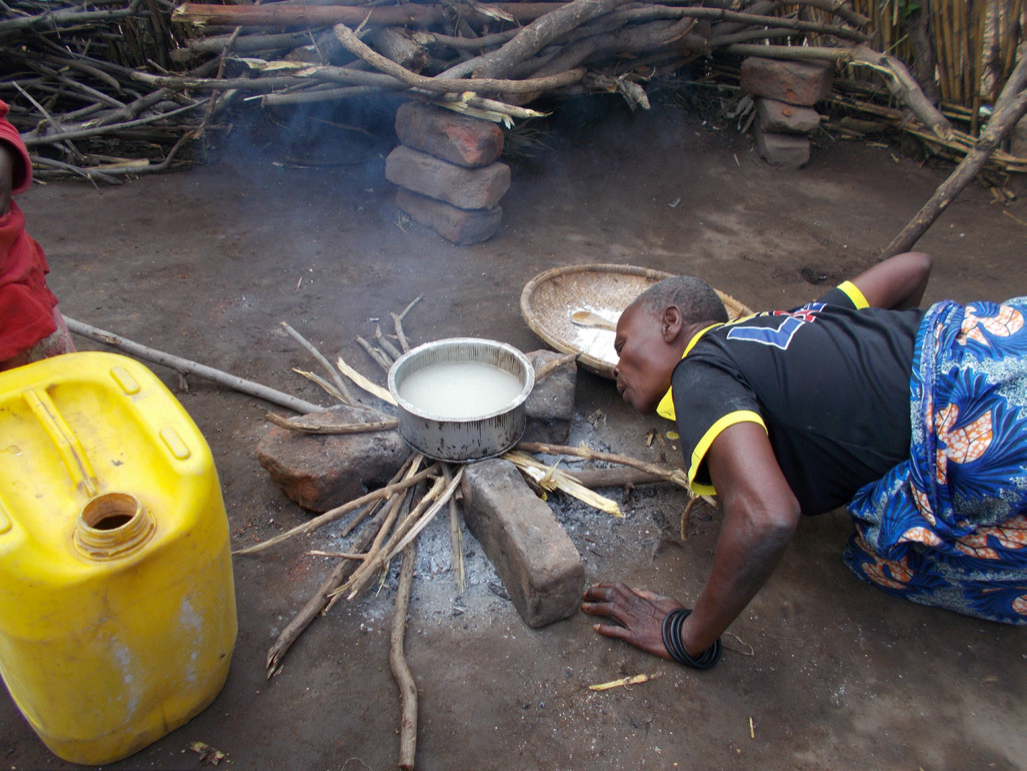
Image of older woman blowing on fire to light it.

When discussing this benefit, respondents also referred to the particular difficulty that elderly, infirm people or pregnant women had when they needed to tend an open fire, as illustrated in figure 4 and described below:

[There was] no need for me to bend or blow air, the cookstove is blowing the air for me up until I gave birth. (female CAPS trial participant Village 1 FGD)

The difficulty described by a CoLT member of convincing CAPS participants to enrol in ancillary studies, indicates how in this context, breath may be seen as something to conserve and linked with bodily strength. These substudies of CAPS involved the temporary installation of household air monitors in homes or spirometry tests. She said those refusing to participate did not ‘think about their health but that you are exhausting their breath’ (CoLT member Village 2). Conversely, cookstoves were therefore valued as cooks did not need to expend breath and strength during use. This direct health benefit to cookstove users only became evident through continued use of the cookstoves and appears to have been an unanticipated aspect of the cookstove both to CAPS participants, workers and researchers. This shows that accessing the expertise of (primarily female) cooks can identify context-specific health drivers of cookstove use.

A second benefit of cookstoves identified by CAPS participants is closely linked to the local context of juggling farming activities with other work and school commitments of families. CAPS trial participants describe how they value cookstoves as they allow food to be cooked more quickly than is possible with open-fire methods. For example, referring to grouped images of different cooking methods in the FGD in Village 1:

On the three stone fire, the food takes a long time to be ready and charcoal stoves make you to be too late because [it needs to] catch fire …while that one which has a fan [the intervention cookstove] …in a short period of time the water is warm, even *nsima* is ready in a short time (female CAPS trial participant Village 1 FGD)

This faster cooking is linked with women having more time for other household responsibilities including farm work and more opportunity for rest. However, this feature is also valued because it facilitates timeliness, that is, it allows cooking to be done or occur within a favourable time. For hungry families who are carrying out hard manual work growing crops on ‘gardens’ not close to their homes, this is a valuable feature. In addition, farming in Chikwawa, whether on a small or larger scale, is often combined with informal, insecure and often arduous work outside the home. As discussed earlier, participants defined good health as having enough to eat, which in turn depended on the ability to participate in precarious livelihood options.

A male COLT member describes this benefit:

[W]e saw that once we are back from the garden, within 10 minutes 20 minutes everything is ready. (CoLT member Village 4)

A female CAPS trial participant suggests that faster cooking is helpful as:

…the food gets prepared so fast that the children do go to school, and the husband does go to work in good time. And it also makes things fast so that if the husband is hungry, in just a little bit of time, if you are cooking beans, it gets cooked in just a little bit of time. (female CAPS trial participant Village 1 FGD)

Overall, time saving through cookstoves is seen as beneficial by CAPS participants as it allows them more free time and to be on time for school and work commitments. Time saved contributes to family harmony for both these reasons, and because it allows hungry people to be fed quickly. Although it has been suggested that faster cooking is linked with time-saving and increased economic opportunities for women and school attendance of girls,^43–45^ the specific benefit of timeliness and the link with household harmony and therefore improved health has not previously been identified.

## Discussion

### Syncretic understandings of health within CAPS

Although biomedicine is the dominant model in global health systems, it is itself a cultural construct related to changing European perceptions of the body in the nineteenth century.^46^ Although medical research and biomedically oriented trials are often beneficial for population health through impacts on health policy and treatment, the pervasiveness of biomedicine, has been linked with the extension of the power of medical professionals and with colonisation.^47^ Kleinman’s influential characterisation of health systems as medically pluralistic, suggests that in most contexts differing medical traditions in the professional, traditional and popular sector coexist.^48^ However, proponents of medical syncretism argue that a binary view of ‘biomedical’ versus ‘lay’ or ‘traditional’ concepts often over-simplifies the ways in which ideas about health are always changing, including the incorporation of biomedical into lay concepts and vice versa. Medical syncretism also places more emphasis on practice as ‘a creative process in which we must recognize the role of invention, innovation, and disorder’.^46^ This is illustrated by Muela *et al* with regard to perceptions of ‘fever’ and ‘malaria’ in a highly researched Tanzanian community, who showed how ‘people are not a “blank slate” but ‘process new information on the basis of what they already know’’.^15^ Similarly, in a study investigating understandings of fever and malaria in the ACTia trial which preceded CAPS in Chikwawa, Ewing *et al* found that the health information from ACTia was intermingled with what was already known, resulting in differing interpretations of fever which in turn impacted on treatment seeking.^49^ The authors of a systematic review of care-seeking for childhood diarrhoea, malaria and pneumonia in sub-Saharan Africa also concluded that there are often considerable differences between how researchers and study participants perceive illness.^50^

The results of this qualitative study suggest that CAPS participant understandings of health were changed through participating in the trial, in that people’s interpretation of trial health messaging created a new linkage between cookstoves and pneumonia. The distinction between comprehension and interpretation made by Downie et al provides a useful way of exploring how these changes occurred. The authors suggest that interpretation is closely linked with the ‘thinking framework’ of the recipient.^51^ CAPS participants were asked to comprehend health messages communicated via the trial and mediated through CoLT members and CAPS fieldworkers. Their interpretation of these biomedical health narratives provided another layer of complexity and the possibility of misunderstandings and unexpected consequences.

It appears that this interpretation included the idea that cookstoves prevent pneumonia. Participant descriptions of why this may be the case were often complicated, and there was some confusion between pneumonia and other bio-medically defined diseases, particularly malaria. It is clear however that this led to health understandings that could potentially have harmful consequences, in the light of the negative findings of the main CAPS trial. That is, it did not lead to a reduction in incidence of pneumonia in under-5s.^9^ Therefore, there was little benefit in associating cookstoves with ‘no pneumonia’, and the potential for harm if such understandings led to delayed treatment seeking for pneumonia or other diseases. More widely, this illustrates the complex challenge of communicating trial objectives to participants and the potential of misinterpretation within a non-biomedical understanding of health.

### The importance of considering local understandings of health

The health and well-being benefits of cookstoves identified by CAPS participants are novel and situated in daily practice and a context of food and livelihood insecurity. They differ from the health benefits of cookstoves as described in the introduction, which are wide-ranging and largely expressed within a biomedical framework. By contrast, the health benefits of saving strength through not blowing, and timeliness improving family harmony, are local and social, linking to wider well-being.^10^ This finding also indicates that researchers and cookstove intervention participants may have differing health priorities. If cookstoves are promoted for their beneficial impact on longer-term health, for example, as by the Malawian cookstove movement Mbaula,^52^ the opportunities may be missed both to produce cookstoves that meet the health needs of users, and that are used more consistently over the longer-term.

Studies that use qualitative methods and participatory methodologies have been shown to provide insight into understandings that bridge biomedical and social aspects of health. For example, Avotri and Walters demonstrated how, in contrast to a common research focus on disease and reproductive health in low-income settings, Ghanaian women emphasised how their productive roles and ongoing financial insecurity led to ‘worrying too much’, headaches and debilitating tiredness.^53^ Similarly, in Bates *et al*’s Photovoice study of the well-being of palliative care patients in urban Malawi, the authors found that ‘emotional well-being, social functioning and contribution’ were key and that ability to work and well-being were closely linked.^54^

The use of the participatory methodology Photovoice in this study allowed a deeper understanding of the potential health and well-being benefits of cookstoves within a specific context. When asked to collect images about the cooking process, Photovoice participants collected and discussed images of growing and processing food and in doing so extended the context of the cooking process beyond the home to encompass all activities referred to by Meah as ‘foodwork’.^55^ That is, they included planting, tending and harvesting crops and the preparation and sale of food, therefore making clear that the local economic environment of insecure farming and other work was linked with ‘foodwork’ and ultimately, cookstove use.^34^

These results demonstrate that the health impacts of the cookstoves as a technological intervention were biosocial, suggesting that it may be important for researchers to pay attention to health as a social construction and interventions as social processes. Through their participation in the trial, cookstove users realised that there was an alternative to open-fire cooking that preserved their breath and energy and allowed them to provide meals quickly for fractious family members. In this way, the possibility of achieving good health as locally defined was enhanced.

### Implications for further research

The results of this study have key implications for further research into clean cooking and for health-based research in low-income settings at the local, national, and global level.

The findings suggest that within MLW and similar research institutions based in low-income settings, the design of further clinical trials and science communication initiatives would benefit from a deeper understanding of how biomedical health information is merged with existing knowledge. In addition, in recognition that new health knowledge that is developed may not always be helpful, researchers would benefit from capturing unanticipated and unintended consequences of understandings of health, through ongoing process analysis of trials.

The unexpected health and well-being benefits ascertained in this study of cookstoves, that is, facilitating timeliness and saving strength through not blowing, have implications at the local and national level. There are ongoing cookstove initiatives in Malawi including the US$1.1. million Malawi Clean Cooking Fund launched in 2020 and various non-governmental projects that could use these findings to maximise the chances that their interventions have a positive impact on health and well-being. That is, through guiding the development of cookstoves that meet the needs of cooks and their families, in terms of usability and enhancing well-being.

Globally, these results imply that health researchers working in low-income settings and particularly the implementors of clean cooking interventions, should prioritise exploration of the locally situated health priorities of participants. This is important to assess whether such interventions meet the priorities of recipients, are beneficial to the target population, and how any benefits should be defined and measured.

At all levels, local, national and global, these results suggest that qualitative methods and the participatory methodology Photovoice can be valuable when exploring complex health understandings. This facilitates in-depth exploration of how knowledge of health is shaped in RCTs and other large-scale interventions and makes a valuable contribution to the interpretation of clinical results and to implementation.^18^

### Strengths and limitations

This was a small-scale qualitative study carried out in a specific highly researched location and generalisation of the results is therefore limited. The Photovoice element of the research was carried out over a short time period which has been identified as a limiting factor when assessing the participatory basis of such research.^56^ As other Photovoice researchers have found, see for example Bates *et al* discussion of the difficulty of visualising discrimination,^54^ the ‘intangible’ concept of ‘health’ required further probing in interviews. In addition, regarding the health perceptions findings, no exploration was carried out prior to the trial, since this change was unintended. However, Photovoice allowed the direct engagement of CAPS participants as researchers who reflected on and communicated the challenges and highlights of their daily lives. The positionality of the first author was considered reflexively throughout the research process and Photovoice was incorporated into the research design to mitigate the outsider status of the first author and any trial-related social desirability bias. Triangulation of sources through including fieldworkers and CoLT members in exploring health perceptions of CAPS participants, enhanced trustworthiness of findings such as the linkage between pneumonia and cookstoves.

The use of qualitative methods combined with Photovoice methodology therefore facilitated a deeper understanding of the health-related concerns of CAPS participants. This highlighted the benefits of qualitative enquiry in foregrounding the health priorities of research participants and enhanced the credibility of the findings.

## Conclusion

CAPS suggested that a cleaner burning biomass-fuelled cookstove intervention alone would not prevent pneumonia in young children in Malawi. However, CAPS trial participants made clear that a link between cookstoves and pneumonia ‘going away’ was established which may have detrimental impacts. This demonstrates the complexities of communicating intended trial outcomes and how these may potentially be misunderstood. The gulf between health understandings of researchers and CAPS participants was also evident from the unexpected contribution to well-being of timeliness and ‘not blowing’. In contexts such as Malawi, cookstoves are likely to continue to be part of the mix of clean energy solutions that will be necessary to achieve the SDGs.^57^ Gaining in-depth understanding through qualitative methods and participatory methodologies can help bridge the divide between cookstove implementors and users^58^ and may ultimately contribute towards the development of approaches that enhance the health and well-being of the billions of people exposed to HAP.

## Data Availability

All data relevant to the study are included in the article or uploaded as supplemental information.
